# Comparison of mammosphere formation from stem-like cells of normal breast, malignant primary breast tumors, and MCF-7 cell line

**DOI:** 10.1186/s43046-022-00152-1

**Published:** 2022-12-12

**Authors:** Jenifer Mallavarpu Ambrose, Vishnu Priya Veeraraghavan, Rosy Vennila, Secunda Rupert, Jeswanth Sathyanesan, Rajasundari Meenakshisundaram, Sakthivel Selvaraj, Sarubala Malayaperumal, Malathi Kullappan, Sudarsanam Dorairaj, Jayesh R. Gujarathi, Sri Harshini Gandhamaneni, Krishna Mohan Surapaneni

**Affiliations:** 1 Department of Research, Panimalar Medical College Hospital & Research Institute, Varadharajapuram, Poonamallee, Chennai, Tamil Nadu 600 123 India; 2 Department of Biochemistry, Saveetha Dental College, Saveetha Institute of Medical and Technical Sciences (SIMATS), Saveetha University, Velappanchavadi, Chennai, Tamil Nadu 600 077 India; 3 Stem Cell Research Centre, Government Stanley Medical College & Hospital, Chennai, Tamil Nadu 600 001 India; 4 PG Research Department of Advanced Zoology and Biotechnology, Loyola College, Chennai, Tamil Nadu 600 034 India; 5 Department of Chemistry, School of Chemical Sciences, KES’s Pratap College, Amalner, Maharashtra 425 401 India; 6 Department of General Medicine, Panimalar Medical College Hospital & Research Institute, Varadharajapuram, Poonamallee, Chennai, Tamil Nadu 600 123 India; 7 Departments of Biochemistry, Molecular Virology, Research, Clinical Skills & Simulation, Panimalar Medical College Hospital & Research Institute, Varadharajapuram, Poonamallee, Chennai, Tamil Nadu 600 123 India

**Keywords:** Breast cancer stem-like cells, Breast cancer stem cells, Mammosphere assay, Mammosphere formation, Mammosphere-forming efficiency

## Abstract

**Background:**

Mammosphere formation assay has become a versatile tool to quantify the activity of putative breast cancer stem cells in non-adherent in vitro cultures. However, optimizing the suspension culture system is crucial to establish mammosphere cultures from primary breast tumors.

**Methods:**

This study aimed at determining the self-renewal and sphere-forming potential of breast cancer stem-like cells derived from human primary invasive ductal carcinoma and normal breast tissue samples, and MCF-7 breast cancer cell line using an optimal suspension culture system. Mammosphere-forming efficiency of the mammospheres generated from the tissue samples and cell line were compared. We evaluated the expression of CD44^+^/CD24^−^/^low^ and CD49f^+^/EpCAM^−^/^low^ phenotypes in the stem-like cells by flow cytometry. CK-18, CK-19, α-SMA, and EpCAM marker expression was assessed using immunohistochemical staining.

**Results:**

Breast epithelial cells isolated from the three samples formed two-dimensional spheroids in suspension cultures. Interestingly, mammospheres formed from patient-derived primary breast tumors were enriched in breast cancer stem-like cells with the phenotype CD44^+^/CD24^−^/^low^ and exhibited a relatively more number of large spheres when compared to the normal breast stem cells. MCF-7-derived SCs were more aggressive and resulted in the formation of a significantly higher number of spheroids. The expression of CK-18/CK-19 and α-SMA/EpCAM proteins was confirmed in breast cancer tissues.

**Conclusions:**

Thus, the use of primary tumor specimens and breast cancer cell lines as suitable models for elucidating the breast cancer stem cell activity was validated using mammosphere culture system.

## Background

Accumulating evidence suggests that cancer originates from a small sub-population of tumor-initiating cells called cancer stem cells (CSCs). Such CSCs possess long-term self-renewing abilities with multipotent differentiating potential, thus giving rise to distinct breast epithelial cell phenotypes within the tumor population [1]. In addition, breast cancer stem cells appear to drive like metastases, recurrence, and chemotherapeutic resistance [2–4]. As CSCs share some of their characteristics with their corresponding normal counterparts [5], understanding their nature and behavior is crucial for improving clinical diagnostics and treatment. Al-Hajj et al. and Dontu et al. reported the presence and activity of breast cancer stem cells (BCSCs) for the first time in 2003 when mammospheres were injected into the mammary fat pad of immunocompromised mice. A minor cell population of cells with CD44^+^/CD24^−/low^/lineage^−^ formed new tumors in their in vivo models [6, 7]. While in vivo transplantation has been considered as a gold standard method to demonstrate the functionality of breast cancer stem cells in animal models [7, 8], mammosphere assay has gained popularity to propagate the normal and cancer stem cells under in vitro culture conditions [9].

Patient-derived primary breast tumors are considered to be a reliable way of studying tumor repopulation using mammosphere assay [10, 11]. However, cell lines are the widely used models to study the human breast cancer stem cell activity as they are easy to handle and cost-effective [12]. Cell lines continue to present the molecular attributes of primary tumors from which they were established [13]. Hence, the present study aimed at exploring the suspension culture system for growing cancer stem-like cell line models from different sources. We present an optimized stem cell culture system to establish the cell lines from fresh primary breast tumor tissues and MCF-7 breast cancer cell line, which is a luminal subtype breast cancer cell line. As a control, mammary stem-like cells isolated from normal breast tissues were compared. The existing stem cell culture techniques were exploited to optimize the culturing methods by comparing the similarities and differences in the growth characteristics of the cell lines obtained from the two sources of breast tumor cells. These in vitro conditions could be adapted to favorably grow cancer stem-like cells from different sources, in suspension cultures for various preclinical studies. Further, the self-renewing ability of the putative stem-like cell populations derived from the three different sources were quantitatively assessed by their mammosphere-forming efficiency (MFE) and cell surface marker expression.

## Methods

### Dissociation of cells from primary breast normal and cancer tissues

This study was ethically approved by the institutional ethics committees of Government Arignar Anna Memorial Cancer Hospital & Research Institute at Kanchipuram, and Stem Cell Research Centre, Govt. Stanley Hospital at Chennai, Tamil Nadu, in compliance with the ethical guidelines framed by the Directorate of Medical Education, Government of Tamil Nadu, India. After obtaining informed written consent, fifteen human breast carcinoma tissue samples were collected from invasive ductal carcinoma patients undergoing mastectomy procedures. Adjacent normal tissues were also obtained 6 cm away from or diagonally opposite to the tumor site on the breast during surgical resection [14]. The tissue specimens were collected in separate containers (normal/tumor) with PBS containing antibiotics (Sigma-Aldrich), transported to the laboratory under sterile conditions, and were processed immediately within 1–2 h.

Fresh tumor and normal tissue samples obtained were processed separately as previously described [10, 14–16]. Briefly, primary tumor breast tissue samples were minced into small fragments of about 1–2 mm^2^, which were subsequently subjected to enzymatic digestion at 37 °C for 16–18 h using 1 mg/ml collagenase type IV and 100 U/ml hyaluronidase type IV-s (Sigma-Aldrich). Similarly, normal tissues, as confirmed by pathological examination, were initially minced into small pieces of 1–2 mm^2^ and then enzymatically digested as mentioned above. Following enzymatic digestion, breast organoids collected by differential centrifugation were further dissociated with trypsin-EDTA and dispase II (Sigma-Aldrich). The dissociated cells were separated by differential centrifugation and prepared for primary mammosphere cultures. Additionally, MCF-7 cell line obtained from NCCS, Pune, India, were initially seeded in a T25 flask containing Dulbecco’s Modified Eagle’s Medium (DMEM/F-12) with 10% FBS (Sigma-Aldrich) and incubated at 37 °C and 5% Co_2_ for further characterization.

### Primary mammosphere culture

The sphere-forming abilities of breast normal and tumor cells derived from patient samples and MCF-7 cell line were evaluated by plating them under low-binding conditions in primary mammosphere culture. To this aim, the cells isolated from the normal tissues, primary tumor tissues, and MCF-7 cell line were separately seeded in triplicate at a density of 1 × 10^5^ cells per well in ultra-low attachment 6-well plates (Corning) containing DMEM-F12 supplemented with 0.2% FBS, 20 ng/mL of human epidermal growth factor (hEGF) (Gibco), 5 ng/mL of insulin (Gibco), 4 ng/mL of heparin (Sigma-Aldrich), 1 μg/mL hydrocortisone (Sigma-Aldrich), and antibiotics. At the time of seeding, 2 mL of mammosphere media was added to each well and the plates were incubated at 37 °C and 5% Co_2_ without disturbing for 5 days, after which 0.5 mL of additional growth medium was added to each well. About a week later, non-adherent compact spheroids of about 50–100 μm in diameter called mammospheres were formed. Mammosphere-forming efficiency of the cells derived from the three sources was calculated by dividing the total number of spheres formed by the total number of viable cells seeded, which was expressed in percentage.

### Mammosphere subculture

The mammospheres of breast normal tissues, primary tumors, and MCF-7 cell line formed in the primary cultures were collected by gentle centrifugation. After generating a single-cell suspension from the spheres, the cells were re-seeded as previously described. Briefly, the stem-like cell-enriched mammospheres collected from the primary mammosphere cultures were centrifuged at 800 rpm for 1 min. After disaggregating the spheroids using 0.25% trypsin-EDTA, the cells were then passed through 40 μm strainers to ensure that a single cell suspension was generated. For serial passaging, breast normal and tumor stem-like cells harvested were re-seeded in triplicate at a limited dilution of 1 × 10^4^ cells per well in ultra-low attachment 6-well plates under non-adherent conditions as described above. Mammospheres of 50 to 100 μm in diameter were counted on days 7, 14, and 21 respectively. Sphere-forming efficiency was calculated at the end of each passage.

### Mammosphere growth in different growth media

The growth of mammospheres was assessed in three different types of growth media, which was prepared with slightly different compositions—(A) DMEM with 10% FBS (serum-supplemented), (B) DMEM supplemented with 0.5 % FBS in addition to growth factors (low serum), and (C) DMEM containing mammary epithelial growth supplements (MEGS) (Gibco) such as pituitary extract, human recombinant insulin-like growth factor, hydrocortisone, and hEGF. Single cells from passage 2 were seeded in triplicate at a density of 1 × 10^4^ cells per well in separate ultra-low attachment plates with 2.5 mL of the three different types of growth media added to each well. The fully grown mammospheres in each medium were subsequently counted and MFE was calculated.

### Flow cytometry

Mammospheres from each passage of the breast normal tissues, primary tumors, and MCF-7 cell line were collected, washed, and stained according to the manufacturer’s protocol. After generating a single-cell suspension, the detached cells were re-suspended in ice-cold PBS with 0.5% FBS at a density of 1 × 10^6^ cells/200 μL. While the stem-like cells obtained from primary tumors and MCF-7 cell line were stained against human CD44-FITC, CD24-PE, the mammosphere-enriched normal cells were incubated with anti-human CD49f-FITC, and anti-human EpCAM-PE (all from BD Biosciences) at 4 °C in the dark for 45 min at recommended concentrations, according to the manufacturer’s instructions. The labeled cells were then washed in PBS to remove any unbound antibodies, analyzed in FACS ARIA-2 (BD Biosciences, USA), and the data were visualized through DIVA software (BD Biosciences, USA). A minimum of 10,000 events was recorded for all samples and the experiments were performed in triplicate.

### Immunohistochemistry

The expression of luminal and myoepithelial markers such as CK-18/CK-19 and α-SMA/EpCAM respectively were evaluated within primary tumor tissues by immunohistochemical staining procedure. Briefly, 4 μm tissue sections were obtained from formalin-fixed and paraffin-embedded blocks of breast tumor tissues. After retrieving the antigens by heat-induced epitope retrieval method, the sections were incubated in anti-CK-18, CK-19, α-SMA, and EpCAM monoclonal antibodies (Biogenex, India) at 4 °C overnight. This was followed by staining with a horseradish peroxidase-conjugated secondary antibody (Biogenex, India) at a dilution of 1:500 in PBS and incubated at room temperature for 1 h according to manufacturer’s instructions. The immunoreactivity was detected by adding diaminobenzidine (DAB) substrate (Biogenex, India). The sections were counterstained, dried, and mounted. Negative controls were stained with the secondary antibody only.

### Statistical analysis

All the data obtained are presented as mean ± standard deviation (SD). Student’s *t* test, analysis of variance (ANOVA), and post hoc Tukey’s tests were performed using SPSS 22.0.0.0 (SPSS Inc., USA). A *P* value < 0.05 was considered as statistically significant to indicate the differences. All the experiments were performed at least three times in triplicate.

## Results

**Fig. 1:**
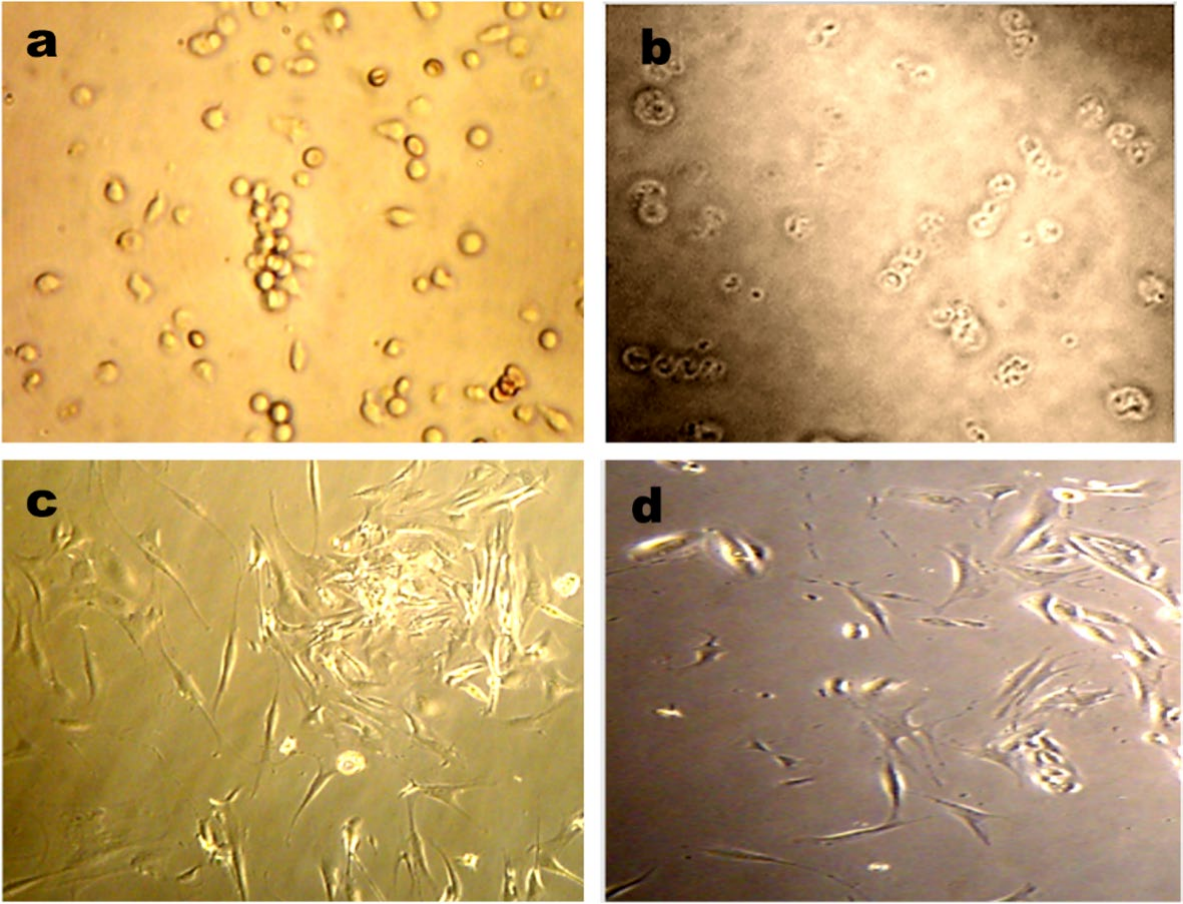
Breast cells obtained from the normal breast tissues, primary breast tumors, and MCF-7 cell line

The age of the experimental subjects was in the range of 47 and 63 years with a mean age of 53.46 ± 4.86 years. Among the fifteen tumor subjects, four tumors belonged to stage 3 (26.67%) and the remaining eleven were of stage 2 (73.33%) tumors. The clinicopathological parameters of the breast cancer subjects are presented in Table 1. It was observed that the tumor tissues yielded a large number of fast-dividing cells while the normal cell cultures yielded a limited number of cells, which were relatively slow in cell division (Fig. 1a–d).

**Table 1:** Clinicopathological parameters of breast cancer subjects

Tumor sample ID no.	Age	Tumor stage	Node status	Neoadjuvant therapy	Family history
IDC001	48	II	N_0_	No	No
IDC002	53	III	N_1_	No	No
IDC003	58	II	N_0_	No	No
IDC004	54	II	N_0_	No	No
IDC005	55	II	N_1_	No	No
IDC008	51	II	N_0_	No	No
IDC009	59	II	N_1_	No	No
IDC010	63	II	N_1_	No	No
IDC011	49	II	N_0_	No	Yes
IDC012	47	II	N_3_	No	No
IDC007	48	III	N_2_	No	No
IDC013	50	II	N_0_	No	No
IDC006	60	III	N_1_	No	No
IDC014	55	II	N_0_	No	No
IDC015	52	III	N_2_	No	Yes

### MFE of stem-like cells isolated from breast normal, primary breast tumor, and MCF-7 cell line

**Fig. 2:**
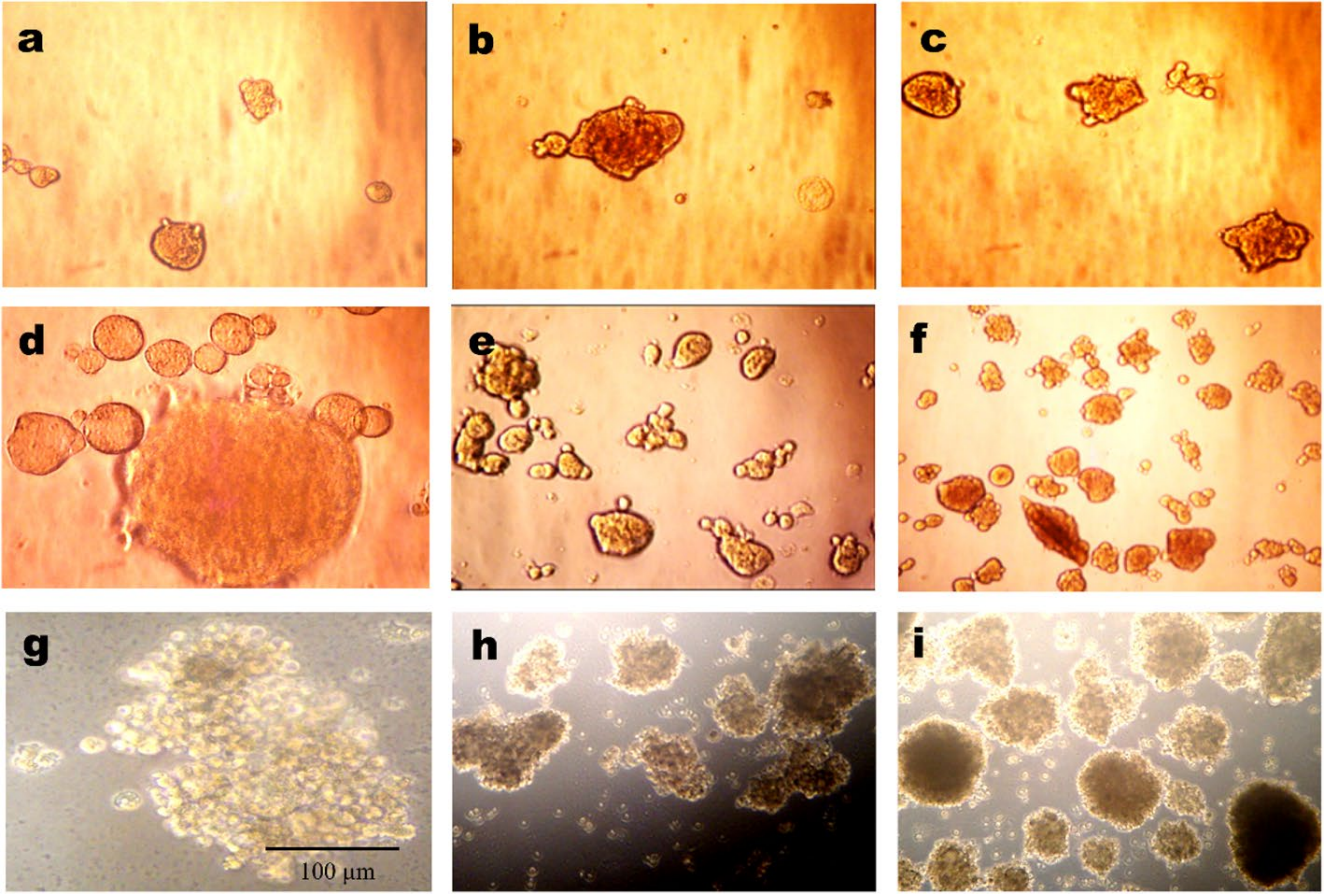
Mammospheres formed from normal breast tissues, primary tumor tissues, and MCF-7 cell line

Putative mammary stem-like cells from the patient-derived tumors, normal breast tissue, and MCF-7 cell line were enriched by mammosphere assay. One week after the initial seeding of the normal breast epithelial cells in suspension culture, a few mammospheres of about 50–70 μm appeared. The spheres formed were irregular in shape with no significant increase in the number of spheres in the subsequent passages; however, their size gradually increased approximately to 70–100 μm in diameter (Fig. 2a–c). The tumor cells in mammosphere culture started forming small-suspended colonies from day 3. A week later, those floating structures increased in size and exhibited more regular spherical and two-dimensional contours. About 10 to 12 mammospheres of 100 μm were formed from the tumor-derived cells. In the second and third generations, an increased number of enlarged mammospheres were developed, which appeared to be 100–150 μm in diameter (Fig. 2d–f). The formation of mammospheres from the MCF-7 cell line began 3 days after initial seeding. The rate of sphere formation was remarkably higher than that of primary tumor cells. Seven days later, more solid and regular spheres of approximately 100–150 μm in diameter were formed (Fig. 2g–i). Some mammospheres displayed an empty bubble-like and loosely clustered cell aggregate morphology in the non-adherent cultures. The number of mammospheres formed per well steadily increased from day 1 to day 7. Furthermore, the cultures of MCF-7 cells beyond the third passage (data not shown) displayed more number of mammospheres per well, with decreased size and dark centers.

**Fig. 3:**
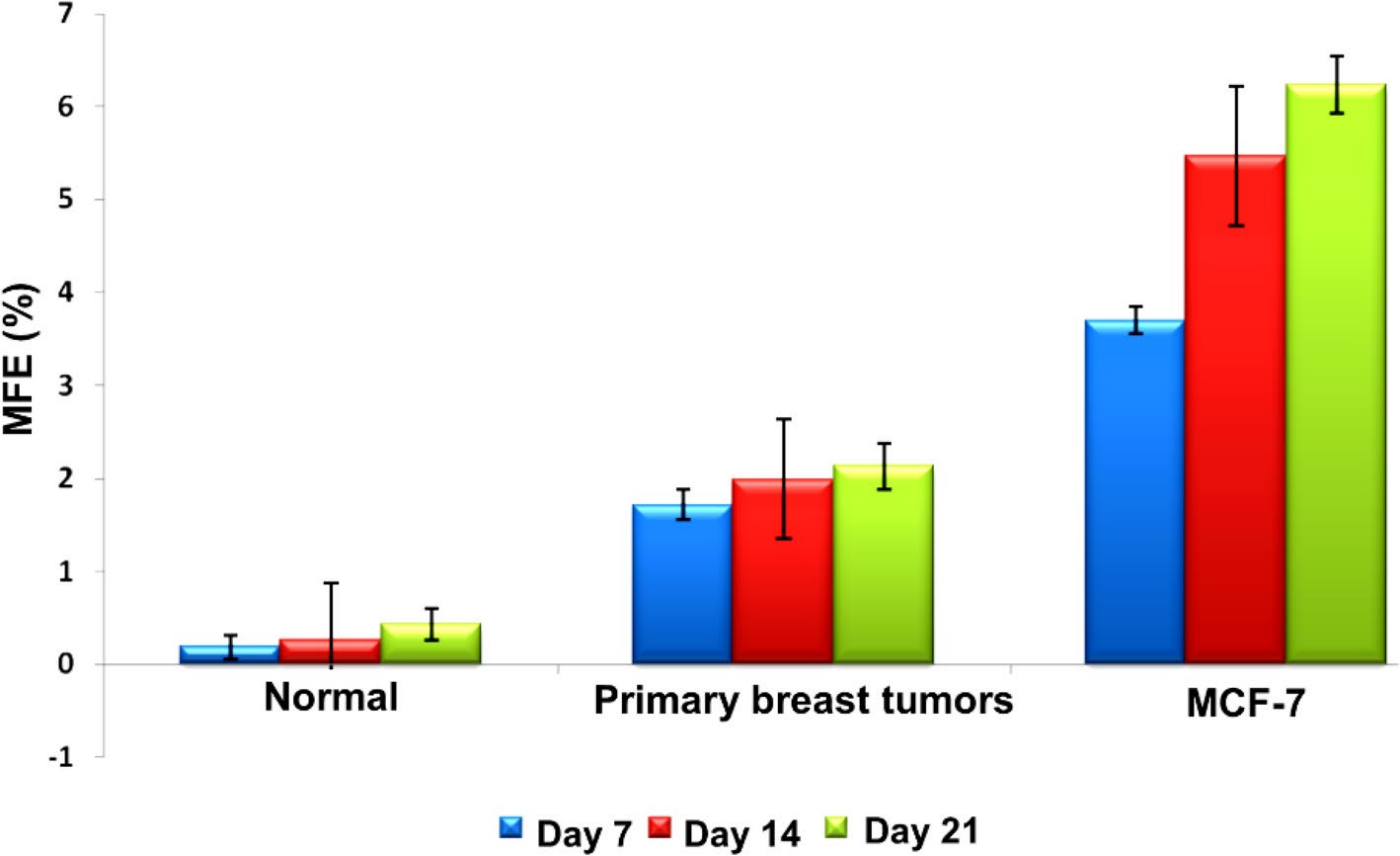
Mammosphere-forming efficiency of stem-like cells isolated from normal breast tissues, primary tumor tissues, and MCF-7 cell line on days 7, 14, and 2

Based on the number of spheres formed after the initial seeding of cells, mammosphere-forming efficiency was calculated at the end of first, second, and third generations in the in vitro cultures. MFE of normal, primary breast tumors, and MCF-7 on day 7 was calculated to be 0.19 ± 0, 1.71 ± 0.62, and 3.70 ± 0.17 respectively with *P* value < 0.001. Similarly, the MFE of the above cells significantly increased at the end of the second and third passages. After 3 weeks of culture, the MFE of primary tumor cells significantly increased to 24.5% on day 21. Likewise, the sphere-forming efficiency of MCF-7 cells remarkably increased to 56.3% (Table 2). The MFE between the groups were statistically compared, which confirmed that there was a significant increase in the MFE of primary tumors, MCF-7 in particular, at the end of days 7, 14, and 21, respectively. The mammosphere-forming efficiency of primary tumors was higher across increasing passages, while the MCF-7 cell line exhibited remarkably a higher MFE among all the three cell types. By contrast, the overall MFE of normal mammospheres was calculated to be very low (Fig. 3).

**Table 2:** Mammosphere-forming efficiency of stem-like cells isolated from normal breast tissues, primary tumors and MCF-7 cell line in increasing passages

S. no.	Mammosphere	Day 7	Day 14	Day 21
1	Normal	0.19 ± 0.13	0.25 ± 0.16	0.43 ± 0.14
2	Primary Tumors	1.71 ± 0.62	1.99 ± 0.64	2.1 ± 0.75
3	MCF-7	3.7 ± 0.17	5.5 ± 0.25	6.2 ± 0.30

### Heterogeneity in the mammospheres formed from three different sources

**Fig. 4:**
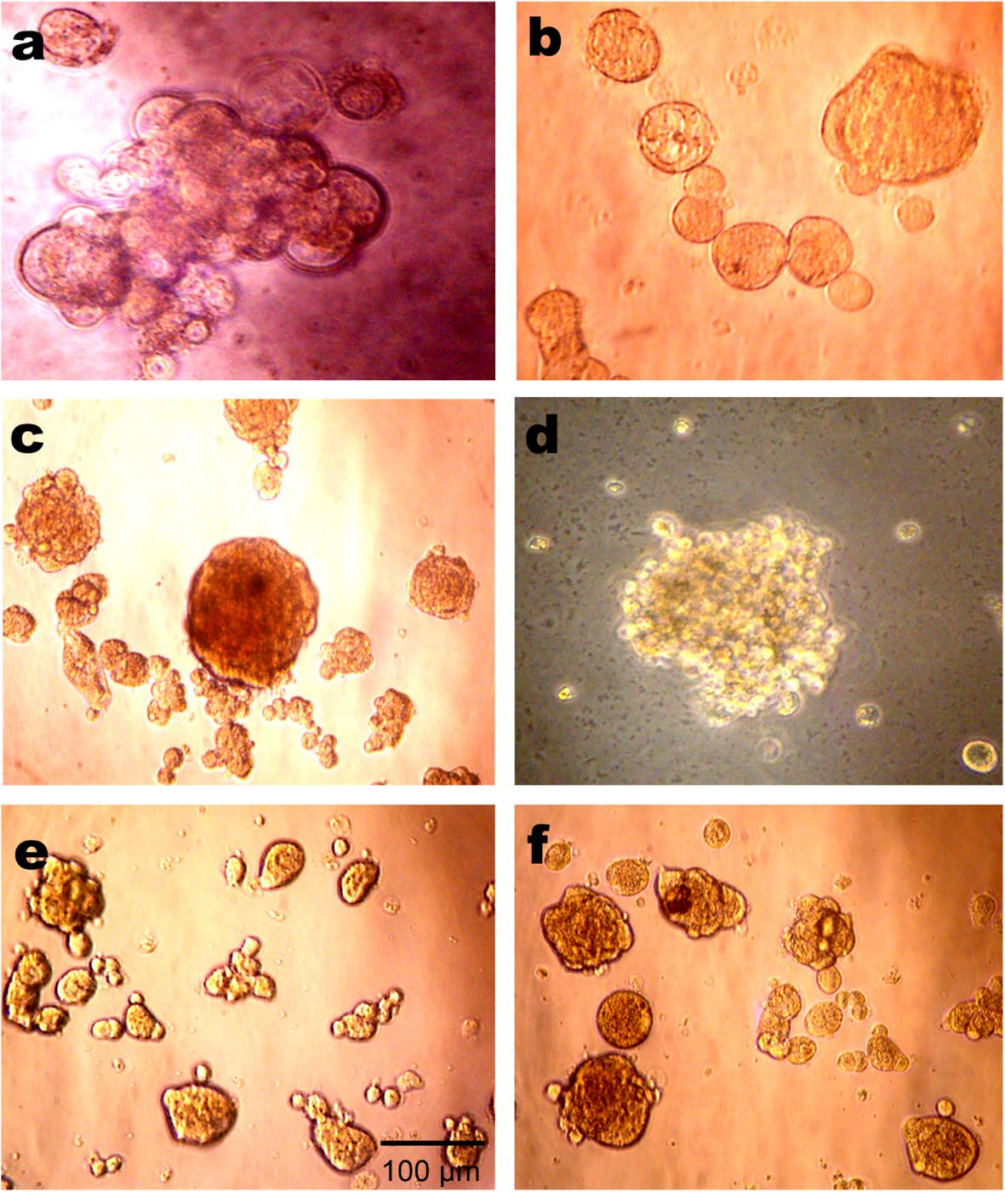
Mammospheres with diverse morphology in mammosphere formation assay

The mammospheres formed from different sources displayed diverse morphologies in the anchorage-independent conditions. The most salient feature of the mammospheres formed was the formation of hollow spheres, in which a few cells were tufted together to form empty bubble-like structures at the centre, surrounded by a few cells or even small such mammospheres bound to those hollow spherical bodies. Another morphology that was observed in the sphere formation assay was the formation of grape-like clusters, in which a bunch of cells was loosely attached. Yet in some cultures, the cells obtained from a few samples formed more solid spheres (Fig. 4a–f).

### Optimal mammosphere culture system

**Fig. 5:**
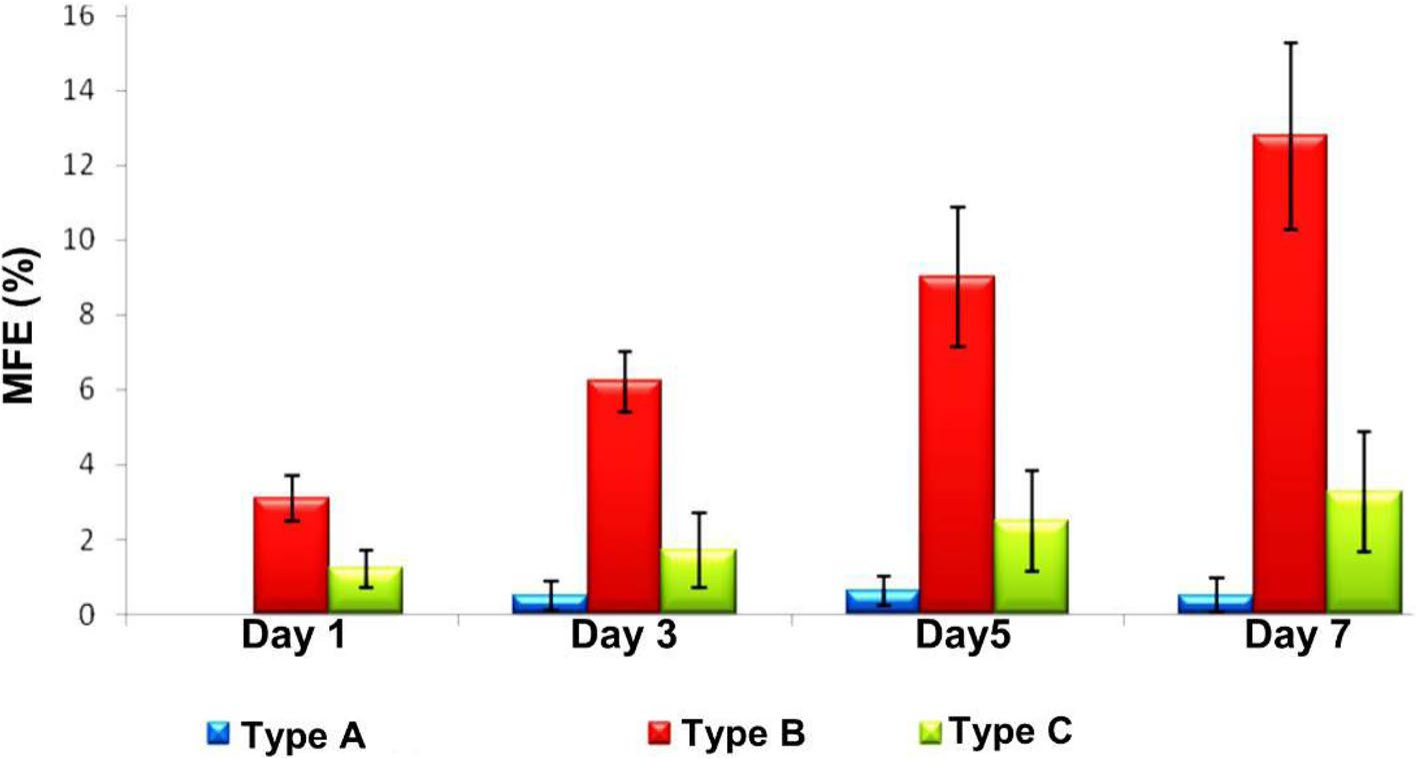
MFE of primary tumor-derived stem-like cells cultured in growth media types A, B, and C

Mammosphere-forming efficiency of the isolated stem-like cells was validated in three different types of mammosphere growth media such as A, B, and C, as mentioned above. It was noteworthy that the primary tumor cells in the culture responded differentially to the three types of growth media mentioned above (Fig. 5). Cells plated in the first growth medium (A) under adherent culture conditions did not promote sphere formation. However, the cells cultured in the second type of medium (B) under low binding culture conditions produced several enlarged mammospheres in the presence of growth factors on day 7 with a mean value of 12.78 ± 2.49 and *P* value < 0.001 (Table 3). Similarly, the cells seeded in the third type of medium (C) supported sphere formation with relatively less number of small mammospheres on day 7, with the mean value of 3.28 ± 1.63and *P* value 0.001.

**Table 3:** Mammosphere-forming efficiency of stem-like cells in growth media A, B, and C

Type	Composition of growth media	Day 1	Day 3	Day 5	Day 7
A	DMEM + 10% FBS (serum supplemented)	0.00 ± 0.00	0.50 ± 0.40	0.61 ± 0.39	.500 ± 0.46
B	DMEM + 0.5% FBS (low serum supplemented)	3.11 ± 0.62	6.22 ± 0.81	9.00 ± 1.86	12.78 ± 2.49
C	DMEM + MEGS (MEGS supplemented)	1.22 ± 0.50	1.72 ± 0.99	2.5 ± 1.35	3.28 ± 1.63

### Expression of CD44^+^/CD24^-/low^ and CD49f^+^/EpCAM^-^ breast cancer/normal stem cell markers

**Fig. 6:**
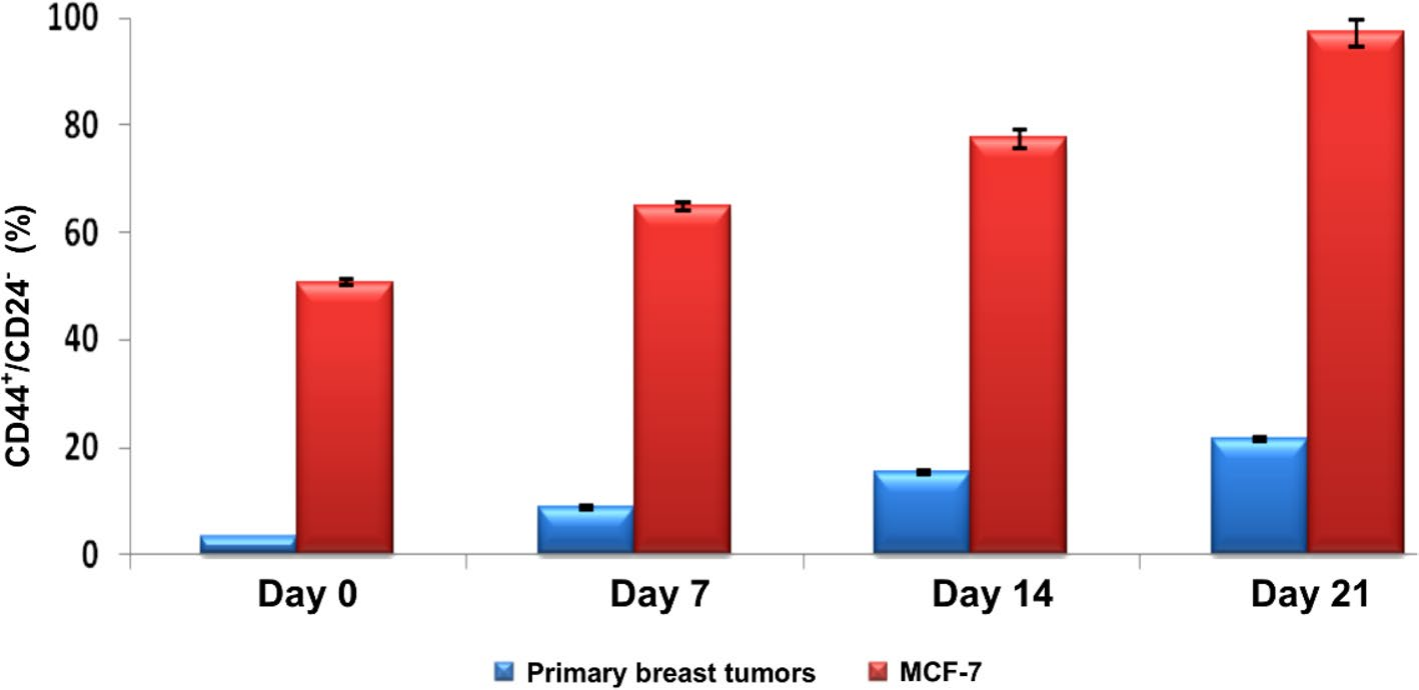
Proportions of cancer stem-like cells with CD44^+^/CD24^−/low^ phenotype from primary breast tumors and MCF-7 cell line on day 7, 14, and 21

The proportions of CD44^+^/CD24^−/low^ and CD49f^+^/EpCAM^−^ cells in the mammospheres from the three different sources were detected in the cells harvested from the non-adherent cultures on days 0, 7, 14, and 21 (Fig. 6). The proportion of primary tumor stem-like cells, which were initially positive for CD44 and negative or weakly positive for CD24 (9.6 ± 2.3%), gradually increased in the second (16.4 ± 1.1%) and third-generation (27.4 ± 9.2%) mammospheres with a significance of *P* < 0.001 (Table 4). Though both primary tumor and MCF-7 cells were of tumor type, the proportion of cells expressing this specific phenotype and their MFE was significantly higher in MCF-7 (*P* < 0.001) cells than that of the primary tumor epithelial cells. MCF-7 cells with the phenotype CD44^+^/CD24^−/low^ formed more aggressive mammospheres with maximum MFE when compared to the mammospheres of primary tumor-derived cells. Furthermore, it was observed that the size of the mammospheres in MCF-7 cells was much larger than that of the primary ductal carcinoma cells. Thus, breast cancer stem-like cells were enriched by culturing MCF-7 and primary breast tumor cells under serum-free conditions in non-adherent culture systems.

**Table 4:** CD44^+^/CD24^−^ stem-like cell population in mammospheres from primary breast tumors and MCF-7 cell line

S. no.	Mammosphere	Day 0 (%)	Day 7 (%)	Day 14 (%)	Day 21 (%)
**1**	Primary tumors	3.6 ± 1.7	9.6 ± 2.3	16.4 ± 1.1	27.4 ± 9.2
**2**	MCF-7	49.0 ± 3.5	63.8 ± 7.3	77.3 ± 6.8	86.5 ± 2.1

**Fig. 7:**
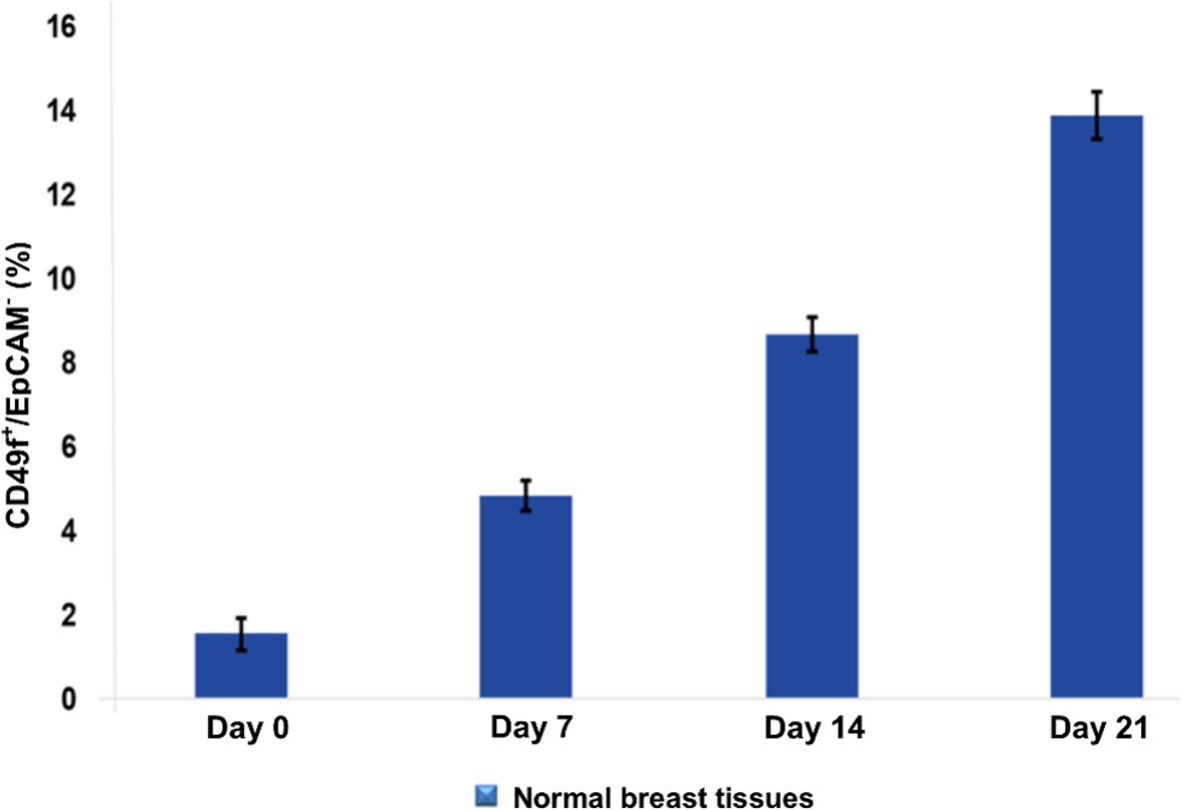
Proportions of normal mammary stem-like cells with CD49f^+^/EpCAM^−/low^ phenotype from normal breast tissue on day 7, 14, and 21

Similarly, the proportion of cells with the immunophenotype CD49f^+^/EpCAM^−^ in the corresponding normal counterparts were analyzed on days 0, 7, 14, and 21, respectively (Fig. 7). In the case of normal breast-derived mammospheres, CD49f^+^/EpCAM^−^ cell proportion in the first passage was 5.4 ± 1.5% (*P* value < 0.001), which subsequently enhanced to 14.5 ± 1.2% (*P* value < 0.001) in the later passage (Table 5). It was evident that the sphere-forming ability of normal stem-like cells was limited and enriching stem cell proportion from the normal source under in vitro mammosphere culture conditions was found to be limited and inconsistent. Thus, the comparative results revealed that the proportion of the stem-like cells obtained from normal breast tissues was limited over that of primary tumors or MCF-7 mammosphere-forming cells.

**Table 5:** CD49f^+^/EpCAM^−^ stem-like cell population in mammospheres from normal breast tissues

S. no.	Mammosphere	Day0 (%)	Day7 (%)	Day14 (%)	Day21 (%)
1	Normal	2.3 ± 2.1	5.4 ± 1.5	9.3 ± 2.5	14.5 ± 1.2

### Immunohistochemistry

**Fig. 8:**
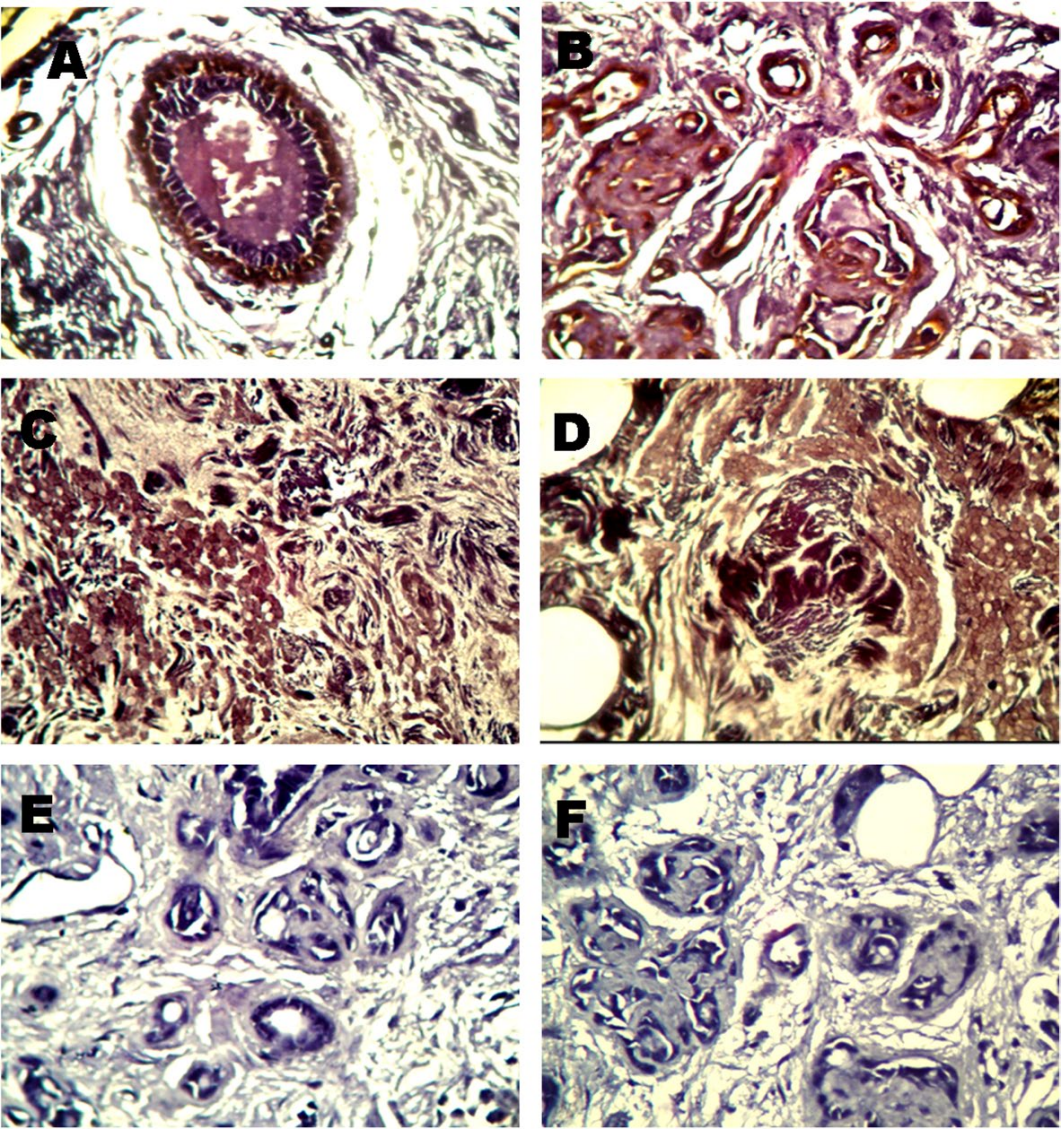
The expression of **a** CK-18, **b** CK-19, **c** α-SMA, and **d** EpCAM in breast carcinoma tissue sections detected by immunohistochemistry

The expression of CK-18/CK-19 (luminal) and α-SMA/EpCAM (myoepithelial) markers were evaluated within primary breast tumor and normal breast tissues studied. The inner layer of luminal epithelial cells in the ducts and acini were positively stained for CK-18 and CK-19. Positivity was indicated by dark brown cytoplasmic and nuclear staining (Fig. 8a, b). Similarly, α-SMA positivity was revealed by the dark-brown cytoplasmic staining. Myoepithelial cells surrounding the ducts, acini and cysts were positively stained. It was observed that the tumor tissue specimens displayed high positivity (Fig. 8c). Likewise, over-expression of EpCAM was observed in the tumor specimens, whose stem-like cell population was correlated with high MFE-forming mammospheres aggressively (Fig. 8d).

## Discussion

In the present study, we successfully established mammosphere cultures from the three sources, whose cells exhibited varying sphere-forming abilities in the mammosphere culture system. In this study, MCF-7 cell line was primarily used because of its significance in representing the majority of invasive human breast cancers that express hormones like estrogen [17]. In contrast to the previous report, MCF-7-derived cells revealed higher tumorigenic potential as the sphere formation was more aggressive with remarkably higher MFE when compared to the primary carcinoma-derived stem-like cells. The number of MCF-7 spheres formed at the end of every passage exponentially increased, which indicated their self-renewing ability. Previous literature has explained that this increase in the number of symmetric self-renewal divisions probably would result in an amplification of the cellular population, by progressively increasing the number of mammospheres at each passage [18–20]. Next to the MCF-7 cell line, the survival and self-renewal of those tumor-initiating stem-like cells of primary tumor tissues were consistent in the mammosphere cultures. On the other hand, the low MFE observed in normal mammosphere cultures demonstrated limited stem cell activity and their quiescent state in maintaining the normal cell state equilibrium. Our findings were in concordance with the previous studies, which reported that low MFE of the stem cells isolated from normal breast tissue suggested that it consists of stem or progenitor cells with limited stem cell activity when compared to breast carcinoma tissue [21]. Apart from size and number, it was noteworthy that the cells in the mammosphere assay tended to develop spheres of a different shape from a regular round shape to lose grape-like clusters to bubble-like hollow spheres to more solid spheres. Spheres with similar morphology have been reported earlier [12]. Our results conclusively demonstrated that mammospheres, rather than being homogeneously enriched with undifferentiated cells, were highly heterogeneous, consisting of morphologically distinct entities with inter and intra-sphere molecular heterogeneity. Thus, the use of the mammosphere assay as a robust tool to demonstrate the peculiar properties of BCSCs was evaluated. Nevertheless, repeatedly applying the same optimized mammosphere culture conditions in the in vitro experiments on a broad range of the available breast cancer cell lines like MDA-MB-231, Hs578T, and Evsa-T might provide consistent and in-depth understanding about the behavior of putative breast cancer stem cells.

Our results also indicated that DMEM supplemented with low serum (0.5% FBS) and growth factors best suits the growth of mammospheres when compared to serum-supplemented or MEGS supplemented medium. The data obtained have confirmed that the spheres achieved the floating tendency in low serum supplemented media and the presence of growth factors such as EGF enhanced the intensity of mammosphere formation in culture. Research studies in the past have confirmed the use of low serum-supplemented medium for establishing successful mammosphere culture systems [22]. The flow cytometry analysis further substantiated the above findings that the putative breast cancer stem-like cells had CD44^+^/CD24^-^ phenotype with moderate to high sphere-forming efficiency in the mammosphere assay. As reported previously, CD44^+^/CD24^−^ cells were prevalent in the tumors, playing a critical role in the invasive step of metastasis [12, 19, 21, 23]. These data suggested that those cells with the CD44^+^/CD24^−^ phenotype were more aggressive in developing mammospheres, thus indicating the higher tumorigenic potential of BCSCs derived from MCF-7 cell line. Similarly, normal mammary stem cells, which displayed low MFE in the mammosphere assay, were CD49f^high^/EpCAM^low^ [22]. In addition, the expression of luminal and myoepithelial markers within primary tumor tissues indicated that the cell types were involved in the mammosphere formation when grown in suspension cultures [24–26].

## Conclusions

Our study demonstrated the optimal approach for establishing stem cell cultures from primary breast tumors in comparison with MCF-7 cell line and normal tissue samples. Under low-serum culture condition in a non-adherent manner, stem-like cells isolated from all the three sources formed mammospheres with varying MFE. MCF-7 showed the highest MFE and tumorigenicity with the cancer stem cell immunophenotype. Therefore, this study validates the use of primary malignant breast tissues, cancer cell line for further in vitro characterization and other molecular investigations on breast cancer stem cells.

## Data Availability

All the data that was generated during the study are available from corresponding author on reasonable request.

## Abbreviations

CSCs: Cancer stem cells
BCSCs: Breast cancer stem cells
MFE: Mammosphere-forming efficiency
DMEM: Dulbecco’s modified Eagle’s medium
hEGF: Human epidermal growth factor
MEGS: Mammary epithelial growth supplements
FBS: Fetal bovine serum
DAB: Diaminobenzidine
CK18/19: Cytokeratin 18/19
